# Co-occurrence of *bla*_*NDM–*1_ and *mcr*-9 in a Conjugative IncHI2/HI2A Plasmid From a Bloodstream Infection-Causing Carbapenem-Resistant *Klebsiella pneumoniae*

**DOI:** 10.3389/fmicb.2021.756201

**Published:** 2021-11-30

**Authors:** Zhou Liu, Xiubing Hang, Xiao Xiao, Wenwen Chu, Xin Li, Yangyang Liu, Xi Li, Qiang Zhou, Jiabin Li

**Affiliations:** ^1^Department of Laboratory Medicine, The Second Hospital of Anhui Medical University, Hefei, China; ^2^Department of Infectious Diseases, The First Affiliated Hospital of Anhui Medical University, Hefei, China; ^3^Anhui Center for Surveillance of Bacterial Resistance, Hefei, China; ^4^Institute of Bacterial Resistance, Anhui Medical University, Hefei, China; ^5^Centre of Laboratory Medicine, Zhejiang Provincial People’s Hospital, People’s Hospital of Hangzhou Medical College, Hangzhou, China; ^6^Department of Infectious Diseases, The Chaohu Hospital of Anhui Medical University, Hefei, China

**Keywords:** *Klebsiella pneumoniae*, carbapenem-resistant, *bla*_*NDM–*1_, *mcr-9*, IncHI2, plasmid

## Abstract

Spread of the carbapenemase-encoding and mobilized colistin resistance (*mcr*) genes among Enterobacteriales poses a great threat to global public health, especially when the both genes are transferred by a single plasmid. Here, we identified a *bla*_*NDM–*1_- and *mcr-9*-co-encoding plasmid harbored by a clinical isolate of *Klebsiella pneumoniae* (KPN710429). KPN710429 was recovered from a blood sample from an inpatient in a tertiary hospital in China, and antimicrobial susceptibility testing showed that it was multidrug-resistant and only susceptible to aztreonam, colistin, and tigecycline. KPN710429 belongs to sequence type (ST) 1308 and capsular serotype KL144. The string test of KPN710429 was negative, and this strain didn’t exhibit a hypervirulent phenotype according to serum-killing and *Galleria mellonella* lethality assessments. Whole-genome sequencing revealed the KPN710429 genome comprises a single chromosome and three plasmids. All virulence associated genes were harbored by chromosome. Most of its antimicrobial resistance genes, including *bla*_*NDM–*1_ and *mcr-9* were carried by plasmid pK701429_2, belonging to the incompatibility (Inc) HI2/HI2A group and ST1. Comparative genomics assays indicates that pK710429_2 could be a hybrid plasmid, formed by a Tn*6696*-like *bla*_*NDM–*1_ region inserting into a *mcr-9*-positive-IncHI2/HI2A plasmid. pK710429_2 contained the conjugative transfer gene regions, Tra1 and Tra2, with some structural variations, and conjugation assays revealed that pK710429_2 was transferable. Although pK710429_2 lacked the *qseB*-*qseC* regulatory genes, *mcr-9* expression was upregulated after pretreatment with colistin for 6 h, leading to colistin resistance in KPN710429. To our knowledge, this is the first report of a *bla*_*NDM–*1_- and *mcr-9*-co-encoding transferable plasmid harbored by a bloodstream-infection-causing *K. pneumoniae* strain in China. Effective surveillance should be implemented to assess the prevalence of the plasmid co-harboring carbapenemase-encoding gene and *mcr-9*.

## Introduction

*Klebsiella pneumoniae* is one of the most notorious members of Enterobacteriales, contributing to both community and nosocomial infections. Rapid spread of carbapenem-resistant *K. pneumoniae* (CRKP) poses a great threat to global public health (Effah et al., 2020). Most CRKP isolates produce carbapenemases, which are grouped into molecular classes A, B, and D. New Delhi metallo-beta-lactamase (NDM)-1, one of the most common class B carbapenemases, hydrolyze carbapenems and other β-lactams except aztreonam (Wu et al., 2019). The *bla*_*NDM–*1_ was first identified in Enterobacteriales from a Swedish patient hospitalized in India in 2008 (Yong et al., 2009). Subsequently, widespread distribution of *bla*_*NDM–*1_-positive Enterobacteriales has been reported worldwide. The *bla*_*NDM–*1_ gene is typically carried by plasmids, which are mostly transferable and co-exist with many other resistance determinants, further complicating clinical anti-infective treatment (Wu et al., 2019). Colistin, a cationic cyclic-peptide, is concern as the last-line therapeutic options for treating severe infections caused by carbapenem-resistant Enterobacteriales, especially *bla*_*NDM–*1_-harboring CRKP (Poirel et al., 2017). However, concerns were further raised regarding the emergence and spread of mobilized colistin-resistance (*mcr*) genes. Since *mcr-1* was initially discovered in China in 2015, a growing number of *mcr*-like genes (from *mcr-2* to *mcr-10*) have been identified worldwide (Liu et al., 2016; Li et al., 2020). Among them, *mcr-9* is a newly emerging variant first identified from a clinical *Salmonella enterica* isolate in the United States in 2019 (Carroll et al., 2019). Notably, co-transfer of *mcr-9* and carbapenemase-encoding genes (e.g., *bla*_*NDM*_, *bla*_*IMP*_, and *bla*_*VIM*_) by a single plasmid has been detected in clinical isolates of *Enterobacter* spp., but not yet reported in *Klebsiella* species (Chavda et al., 2019; Li et al., 2020; Ai et al., 2021).

In this study, we characterized the genomic features of a *bla*_*NDM–*1_- and *mcr-9-*co-encoding plasmid harbored by a clinical CRKP isolate. These findings will supplement existing data on the molecular epidemiological characteristics of plasmids co-harboring carbapenemase-encoding gene and *mcr-9.*

## Materials and Methods

### Strain Identification

The *K. pneumoniae* strain KPN710429 was isolated from a blood sample from a 69-year-old woman with duodenal cancer at the Second Hospital of Anhui Medical University in Anhui, China, in June 2020. This bloodstream infection was hospital-acquired, and the patient was not treated with polymyxin B or colistin before this strain was isolated. The species was identified using matrix-assisted laser desorption ionization time-of-flight mass spectrometry (MALDI-TOF-MS) (Bruker, Bremen, Germany).

### Antimicrobial Susceptibility Testing

*In vitro* antimicrobial susceptibility tests (ASTs) were performed for amikacin, aztreonam, cefepime, cefotaxime, cefoxitin, ceftazidime, ceftazidime-avibactam, ceftriaxone, cefuroxime, ciprofloxacin, colistin, ertapenem, gentamicin, imipenem, levofloxacin, meropenem, piperacillin-tazobactam, ticarcillin-clavulanic acid, tigecycline, and tobramycin using the broth microdilution method. The susceptibility breakpoints of tigecycline and colistin were interpreted in according to the European Committee on Antimicrobial Susceptibility Testing (EUCAST) breakpoint (EUCAST, 2020); all other antimicrobial agents were interpreted using Clinical and Laboratory Standards Institute (CLSI) guidelines (CLSI, 2020). The standard *Escherichia coli* strain ATCC25922 was used for quality control.

### Hypermucoviscous Phenotype Detection and Virulence Assessments

The hypermucoviscous phenotype was detected using the string test as described previously (Mazloum et al., 2016). The virulence of KPN710429 were assessed by serum-killing and *Galleria mellonella* lethality assays according to previously described (Liu et al., 2021). For references, a hypervirulent *K. pneumoniae* (designated as KPN54798*^H–ctrl^*) and classic *K. pneumoniae* (designated as KPN49*^L–ctrl^*) were employed as hypervirulence and low-virulence control, respectively (Liu Z. et al., 2019b).

### Whole-Genome Sequencing, Assembly, and Comparative Analysis

The total genomic DNA of strain KPN710429 was extracted using the QIAamp DNA Mini Kit (Qiagen, Hilden, Germany). Whole-genome sequencing (WGS) and assembly were performed as previously described (Liu et al., 2021). Virulence associated genes were predicted using VFDB^1^ (Liu B. et al., 2019a). Antimicrobial resistance genes (ARGs) were identified by using Resfinder^2^ (Bortolaia et al., 2020). The multilocus sequence type (MLST) was identified by submitting the genome sequence to MLST 2.0 (Larsen et al., 2012). Capsular serotyping was achieved by submitting the genome sequence to Kaptive Web^3^ (Wyres et al., 2016). Each plasmid identified in this study was analyzed using PlasmidFinder^4^ and pMLST^5^ to investigate the replicons (Carattoli et al., 2014), and sequences were compared using BLAST analysis and the BLAST Ring Image Generator (Alikhan et al., 2011). Sequence alignments for the genetic environments of *bla*_*NDM–*1_ and *mcr-9* were performed using BLAST and visualized with Easyfig v 2.2.3 (Sullivan et al., 2011).

### Conjugation Experiments

The transferability of the *bla*_*NDM–*1_ and *mcr-9* genes was assessed using conjugation as previously described (Ai et al., 2021). Approximately 1 × 10^8^ colony-forming units (CFU)/mL of the donor strain (KPN710429) and the recipient strain (sodium azide-resistant *E. coli* J53) were mixed at 1:1 ratio and spotted onto filter membrane, which were placed on Muller-Hinton (MH) agar plates and incubated for 18–24 h at 37°C. Transconjugants were selected on MH agar containing both sodium azide (300 μg/mL) and ceftriaxone (32 μg/mL). Presence of *bla*_*NDM–*1_ and *mcr-9* in the transconjugants (designated as J53-KPN710429) was confirmed by PCR and sequencing analysis, Supplementary Table 1 lists the primers sequences. The minimum inhibitory concentrations (MICs) of antimicrobial agents for the transconjugants were assessed using the broth microdilution method.

### Colistin Induction Assays

Colistin induction assays were performed as previously described with some modifications (Kieffer et al., 2019). KPN710429 were inoculated in MH broth supplemented with colistin (0.5, 1.0, and 2.0 μg/mL) or without colistin at final bacterial suspensions of 1.0 MCF, respectively. After shaking at 37°C for 6 h, the strains were enriched by centrifugation and washed three times with sterile saline, then 0.5 MCF bacterial suspensions were prepared for colistin susceptibility testing and mRNA extraction. The relative expression level of *mcr-9* and IncHI2-*repA* were determined by quantitative real-time (qRT)-PCR as described previously (Pfaffl, 2001). Expression of the *K. pneumoniae rpoB* housekeeping gene was used to normalize the transcript levels.

Additionally, the *mgrB* gene of induced strains was amplified and sequenced as previously described (Haeili et al., 2017), then the above sequences were aligned with that of the original strain to determine whether the *mgrB* was mutated during the colistin induction assay. Supplementary Table 1 lists the primers sequences.

### Statistics

The Shapiro–Wilk method was used to test normality. Normally data are presented as means ± standard deviations. Bliss method were used to calculated the 50% lethal dose (LD_50_) of *G. mellonella* larvae (72 h after infection), and the LD_50_ values were expressed as log_10_(lg) transformed values. Statistical analysis was performed with GraphPad Prism (San Diego, CA, United States) using unpaired Student’s *t*-tests. *P*-values of <0.05 was considered statistically significant.

### Nucleotide Sequence Accession Numbers

The *K. pneumoniae* KPN710429 genome sequence was submitted to GenBank under accession numbers CP073657 (plasmid pK710429_1), CP073658 (plasmid pK710429_2), CP073659 (plasmid pK710429_3), and CP073660 (chromosome of KPN710429).

### Ethics Statement

The *K. pneumoniae* strain KPN710429 was isolated from a clinical specimen, generated as part of routine clinical laboratory procedure. The Ethics Committee of The Second Hospital of Anhui Medical University exempted this study from review because it focused only on bacteria.

## Results

### Antimicrobial Susceptibility and Virulence Assessments

Strain KPN710429 was identified as *K. pneumoniae* using MALDI-TOF-MS, and its string test was negative. *In vitro* AST results showed that strain KPN710429 was resistant to all cephalosporins, carbapenems, β-lactam/β-lactamase inhibitors, aminoglycosides, and quinolones, but susceptible to aztreonam, tigecycline and colistin (Table 1). The serum-killing assays demonstrated that KPN710429 was serum intermediate sensitive (grade 3) (Supplementary Figure 1). The lgLD_50_ of *G. mellonella* larvae due to KPN710429 (5.54 ± 0.16) was higher than that due to KPN54798*^H–ctrl^* (4.93 ± 0.08) (*P* = 0.0043), and there was no significant different from that due to KPN49*^L–ctrl^* (5.86 ± 0.19) (*P* = 0.0907).

**TABLE 1:** Antimicrobial susceptibility of strain KPN710429, its transconjugants and *E. coli* J53.

Antimicrobial agents		KPN710429	J53-KPN710429	*E. coli* J53
		MIC*^a^*	MIC*^a^*	MIC*^a^*
Aminoglycosides	AMK	64(R)	32(*I*)	2(S)
	GEN	64(R)	8(*I*)	1(S)
	TOB	64(R)	8(*I*)	1(S)
Monocyclic β-lactam	ATM	1(S)	1(S)	1(S)
Cephalosporin	FEP	64(R)	16(R)	0.125(S)
	FOX	128(R)	64(R)	2(S)
	CAZ	128(R)	64(R)	0.125(S)
	CRO	128(R)	64(R)	0.25(S)
	CXM	128(R)	64(R)	0.25(S)
	CTX	128(R)	64(R)	0.25(S)
β-lactam/β-lactamase inhibitor	TIC	128(R)	64(R)	1(S)
	TZP	128(R)	128(R)	1(S)
	CZA	64(R)	32(R)	1(S)
Carbapenems	ETP	32(R)	16(R)	0.125(S)
	IPM	32(R)	8(R)	1(S)
	MEM	32(R)	8(R)	0.25(S)
Quinolones	CIP	4(R)	0.5(*I*)	0.0625(S)
	LEV	8(R)	1(*I*)	0.125(S)
Others	TGC	0.5(S)	0.5(S)	0.5(S)
	COL	1(S)	1(S)	0.5(S)

*^a^The unit for MIC is μg/mL. MIC, minimum inhibitory concentration; S, susceptible; I, intermediate; R, resistant; AMK, amikacin; ATM, aztreonam; FEP, cefepime; FOX, cefoxitin; CAZ, ceftazidime; CZA, ceftazidime-avibactam; CRO, ceftriaxone; CTX, cefotaxime; CXM, cefuroxime; CIP, ciprofloxacin; COL, colistin; ETP, ertapenem; GEN, gentamicin; IPM, imipenem; LEV, levofloxacin; MEM, meropenem; TIC, ticarcillin-clavulanic acid; TZP, piperacillin-tazobactam; TGC, tigecycline; and TOB, tobramycin.*

### Genome Feature of *K. pneumoniae* Strain KPN710429

Whole-genome sequencing revealed that the genome of *K. pneumoniae* strain KPN710429 comprised a single chromosome (5,124,764 bp) and three plasmids ranging in size from 70,448 bp to 288,244 bp (Table 2). *In silico* analysis assigned this isolate to sequence type (ST) 1308 and serotype KL144. ResFinder analysis was performed to identify a number of ARGs. Among them, *bla*_*OKP*_, *fosA*, *oqxA*, and *oqxB* were located on the chromosome, while all other ARGs, *aac (6′)-Ib-cr*, *aadA2b*, *ant (2″)-Ia*, *bla*_*NDM–*1_, *mcr-9*, *qacE*, *qnrA1*, *sul1*, and *tet(A)*, were harbored by the largest plasmid pK710429_2. Virulence gene analysis showed that the aerobactin receptor gene (*iutA*), enterobactin gene cluster (*entABCDEFS*/*fepABCDG*), salmochelin gene cluster (*iroEN*), type 1 fimbriae gene cluster (*fimABCDEFHIK*), type 3 fimbriae gene cluster (*mrkABCDFHIJ*), were present on the chromosome, while no virulence associated genes were detected in the three plasmids.

**TABLE 2:** Genomic features of the strain KPN710429.

Parameter	Chromosome	pK710429_1	pK710429_2	pK710429_3
Genome size (bp)	5,124,764	170,592	288,244	70,448
GC content (%)	58.05	51.67	46.91	52.44
Plasmid Inc. type	NA	IncFIB	IncHI2/HI2A	IncR
MLST/pMLST	ST1308	None	ST1	None
Antimicrobial resistance gene	*bla*_*OKP*_, *fosA*, *oqxA*, *oqxB*	None	*aac (6′)-Ib-cr, aadA2b*, *ant (2″)-Ia*, *bla*_*NDM–*1_, *mcr-9*, *qacE*, *qnrA1*, *sul1*, *tet(A)*	None
Virulence gene	*entABCDEFS*, *fepABCDG*, *iutA*, *iroEN*, *mrkABCDFHIJ*, *fimABCDEFGHIK*	None	None	None

*Inc, incompatibility; NA, not applicable.*

### Comparative Genomic Analysis of pK710429_2 and Conjugation Experiment

**FIGURE 1:**
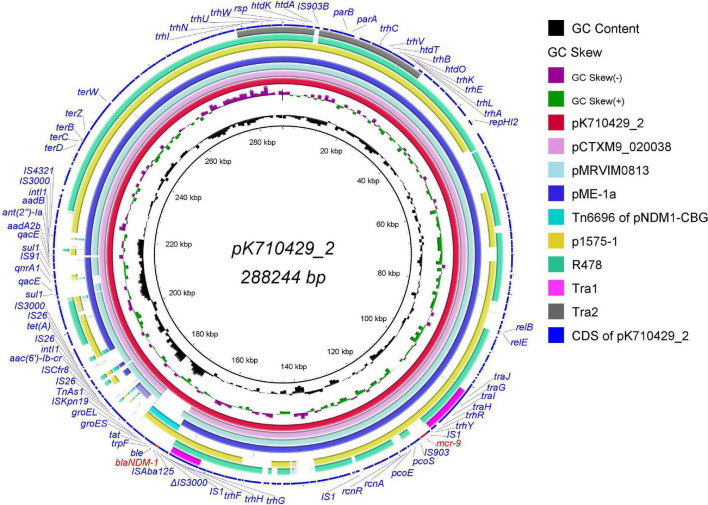
Genomic sequence of plasmid pK710429_2 compared with other IncHI2 plasmids, the Tn6696 region and the Tra1/Tra2 region. The complete plasmid pK710429_2 sequence was used as the reference, and the white and colored regions of the circles indicate absence and presence, respectively. The circles from inside to outside indicate the GC content of plasmid pK710429_2, GC skew of plasmid pK710429_2, plasmid pK710429_2, plasmid pCTXM_020038 (accession no. CP031724), plasmid pMRVIM0813 (accession no. KP975077), plasmid pME-1a (accession no. CP041734), the Tn6696 region of plasmid pNDM1-CBG (accession no. CP046118), plasmid p1575-1 (accession no. CP068288), plasmid R478 (accession no. BX664015), the Tra1/Tra2 region in R478 and coding sequence (CDS) of plasmid pK710429_2. *bla*_*NDM–1*_ and *mcr-9* are indicated in red; the other genes are in blue. Δ indicates the truncated gene.

Sequence analysis showed that pK710429_2 was a large multidrug resistant (MDR) plasmid with a length of 288,244 bp and containing 302 coding genes. This plasmid belongs to the IncHI2/HI2A group with an average G + C content of 46.91%. With 81% query coverage and 99.97% identity in BLASTn, the sequence of pK55602_2 was partially consistent with that of plasmid p1575-1 (accession no. CP068288), a recently reported *bla*_*NDM–*1_-*mcr-9*-co-positive-IncHI2 plasmid harbored by a clinical isolate of *Enterobacter hormaechei* (Ai et al., 2021; Figure 1). Further analyses revealed that the pK710429_2 sequence can be roughly divided into two distinct regions based on its similarity to different plasmids. The first *mcr-9*-harboring-region (accounted for 97% of the plasmid sequences) was highly similar to the sequences of several previously reported *mcr-9*-harboring-plasmids, including pCTXM9_020038 (99% query coverage and 99.99% identity, accession no. CP031724), pMRVIM0813 (98% query coverage and 99.99% identity, accession no. KP975077), and pME-1a (96% query coverage and 99.99% identity, accession no. CP041734). Seven ARGs [*aac(6′)-Ib-cr*, *aadA2b*, *ant(2″)-Ia*, *qacE*, *qnrA1*, *sul1*, and *tet(A)*] and two class 1 integrons were found clustered within a 39,671-bp-long array of IS*26*/IS*4321*-bounded gene cluster (positions 168, 917-176, 764 bp) in this region. The second *bla*_*NDM–*1_-harboring-region, accounted for 3% of the plasmid sequences (positions 168, 917-176, 764 bp), was highly similar to the sequences of the Tn*6696* region in plasmid pNDM1-CBG (100% query coverage and 99.99% identity, accession no. CP046118) (Figure 1).

Additionally, with 76% query coverage and 99.96% identity in BLASTn, the sequence of pK55602_2 was partially consistent with that of plasmid R478 (accession no. BX664015), the first prototype IncHI2 plasmid in which all the conjugative transfer genes were present in the Tra1 and Tra2 regions (Gilmour et al., 2004). Different from plasmid R478, the Tra1 region in pK710429_2 was divided into two parts at 101,547–111,838 bp and 161,470–167,880 bp, and the Tra2 region was split by an IS*903B*-like element (Figure 1). In the conjugation experiments, pK710429_2 was transferred to *E. coli* J53 at the frequency of 1.2 × 10^–6^. PCR detection confirmed that the transconjugant (J53-KPN710429) was positive for *bla*_*NDM–*1_ and *mcr-9*. Phenotypic testing of the transconjugant showed that it was resistance to all β-lactams except aztreonam (Table 1).

### Genetic Environment of *bla*_*NDM–*1_ and *mcr-9*

**FIGURE 2:**
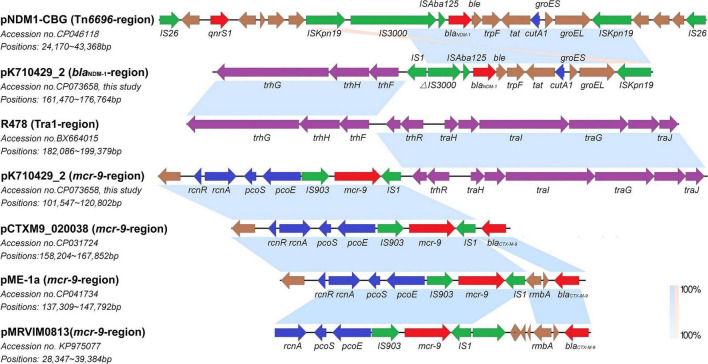
Co-linear analyses for the genetic environment of *bla*_*NDM–1*_ and *mcr-9* in plasmid pK710429_2. The *bla*_*NDM–1*_-harboring-region in plasmid pK710429_2 was compared with TN*6696*-region in plasmid pNDM1-CBG and Tra1-region in plasmid R478; the *mcr-9*-harboring-region in plasmid pK710429_2 was compared with Tra1-region in plasmid R478, *mcr-9*-harboring-region in plasmid pCTXM9_020038, pME-1a, and pMRVIM0813. Blue or orange shading denotes regions of shared homology or inversion among different plasmids, respectively. Colored arrows indicate open reading frames, with red, green, blue, purple, and brown arrows representing antimicrobial resistance genes, mobile elements, heavy metal resistance genes, conjugative transfer genes, and plasmid backbone genes, respectively. Δ indicates the truncated gene.

In plasmid pK710429_2, *bla*_*NDM–*1_ gene was located on a region with the cassette structure of ΔIS*3000-*IS*Aba125-bla*_*NDM–*1_*-ble-trpF-tat-cutA1-groES-groEL-*IS*Kpn19*, which was partly similar to that of Tn*6696* in pNDM1-CBG. Parts of the Tar1 region were located upstream of this cassette, with an IS*1* between them. Interestingly, the remaining parts of the Tar1 region were located downstream of *mcr-9*, also with an IS*1* between them. The *rcnR-rcnA-pcoE-pcoS-IS903* structure, locating upstream of *mcr-9* in pK710429_2, was consistent with that of plasmid pCTXM9_020038, pME-1a, and pMRVIM0813 (Figure 2). However, *qseB*-*qseC* regulatory genes were not found in plasmid pK710429_2.

### Colistin Induction Assays

**FIGURE 3:**
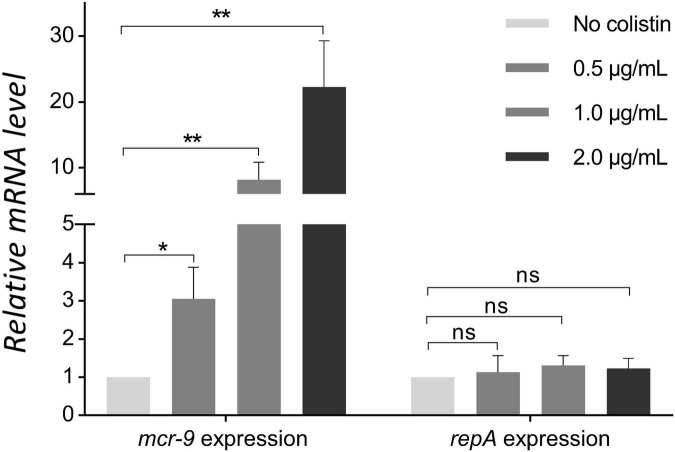
Comparison of *mcr-9* and *repA* expression levels in strain KPN710429 with and without colistin pretreatment. Error bars indicate the standard deviation for three triplicate samples. Asterisks indicate statistical significance at different levels by Student’s *t*-tests. *P < 0.05; **P < 0.01; ns, not significant.

The inducibility of *mcr-9* expression was detected using colistin induction assays. After pretreatment with 0.5, 1, and 2 μg/mL of colistin, the KPN710429 exhibited colistin MICs of 4, 16, and 32 μg/mL, respectively. The *mcr-9* gene expressions were upregulated accordingly, while IncHI2 plasmid replicase gene *repA* expression remained unchanged (Figure 3). The *mgrB* gene was not mutated during the colistin induction assay (Supplementary Figure 2).

## Discussion

Few clinical anti-infective therapeutic options exist to combat the emergence and spread of MDR gram-negative pathogens co-harboring carbapenemase-encoding and *mcr*-family genes (Mmatli et al., 2020). Although *mcr-9* was first identified in 2019, recent research indicates that it has existed for a long time and has been spreading among CRKP isolates of human origin in Europe since 2013 (Wang et al., 2020). In China, clinical isolates of Enterobacteriales co-harboring *bla*_*NDM–*1_ and *mcr-9* have been reported, all of which belong to *Enterobacter* spp., and the *bla*_*NDM–*1_ and *mcr-9* genes were harbored by different plasmids in most strains (Yuan et al., 2019; Lin et al., 2020; Ai et al., 2021). Here, we report a *bla*_*NDM–*1_ and *mcr-9* co-harboring clinical isolate of *K. pneumoniae*, with both *bla*_*NDM–*1_ and *mcr-9* located on the same plasmid. To our knowledge, this is the first report of a *bla*_*NDM–*1_ and *mcr-9* co-encoding plasmid harbored by clinical CRKP isolate in China.

Carbapenem-resistant *K. pneumoniae* strain KPN710429 belongs to ST1308, a rarely sequence type which has been reported as a singleton clone and recovered from inanimate surfaces in hospital environments (Zenati et al., 2017). The string test of KPN710429 was negative, and this strain didn’t exhibit a hypervirulent phenotype according to *in vitro* and *in vivo* virulence assessments. AST results and the international classification scheme (Magiorakos et al., 2012) classify strain KPN710429 as an MDR bacterium, with an MDR phenotype closely related to the ARGs it harbored. WGS data revealed that most ARGs, including *bla*_*NDM–*1_ and *mcr-9*, were harbored by the IncHI2/HI2A-ST1 plasmid, pK710429_2. Notably, IncHI2-ST1 plasmids were found to be the predominant replicon type carrying *mcr-9*, and the *rcnR-rcnA-pcoE-pcoS-IS903-mcr-9-wbuC* structure was consistent in most *mcr-9* cassettes (Li et al., 2020). pK710429_2 also harbored this core structure but without *wbuC*. Additionally, no *qseC-qseB*, a two-component system involved in *mcr-9* expression (Kieffer et al., 2019), was found in pK710429_2, which might explain the colistin susceptibility phenotype of strain KPN710429. Further analysis indicated that pK710429_2 was a conjugative plasmid, and pK710429_2 contained the conjugative transfer gene regions, Tra1 and Tra2, with some structural variations.

In addition to the conjugative plasmid, the accumulation and dissemination of resistance genes was largely due to the actions of mobile genetic elements, including insertion sequences, transposons, gene cassettes, and integrons. In this study, the cassette structure harboring *bla*_*NDM–*1_ was flanked by several mobile elements. Notably, the *bla*_*NDM–*1_ cassette was similar to that of Tn*6696*, a recently reported transposon (Chen et al., 2020), suggesting that the plasmid pK710429_2 could be a hybrid plasmid, formed by a Tn*6696*-like *bla*_*NDM–*1_ region inserting into a *mcr-9*-positive-IncHI2/HI2A plasmid.

Unlike other alleles of *mcr* genes which consistently display colistin resistance, most of the *mcr-9*-positive isolates were found to susceptible to colistin (Carroll et al., 2019; Chavda et al., 2019; Tyson et al., 2020). The *mcr-9* is an inducible gene encoding phosphoethanolamine transferase, and *mcr-9* expression was induced in the presence of colistin (Kieffer et al., 2019). In this study, the colistin resistance phenotype remained absent in KPN710429, but the induction assays showed that after pretreatment with different concentrations of colistin for 6 h, KPN710429 showed the colistin resistance phenotype, and the *mcr-9* gene expressions were upregulated accordingly. Because mutation of *mgrB* gene is one of the mechanisms that contributes to colistin resistance in *K. pneumoniae* (Haeili et al., 2017), we further confirmed that no *mgrB* mutation occurred in KPN710429 during the colistin induction assay. A previous study revealed that the lacking of two-component regulatory genes *qseC-qseB* was commonly observed among the *mcr-9*-positive plasmid (Li et al., 2020). In this study, plasmid pK710429_2 also lacked the *qseB-qseC* regulatory genes. More research is needed to confirm the essential role of the *qseC*-*qseB* module or the involvement of other genes in *mcr-9* induction.

Additionally, the novel antibiotic ceftazidime-avibactam was ineffectively against *bla*_*NDM–*1_-positive bacteria (Zasowski et al., 2015), thus, clinical CRKP isolates co-harboring *bla*_*NDM–*1_ and *mcr-9* usually exhibit a colistin sensitive but ceftazidime-avibactam resistant phenotype, which may mislead colistin to become a very possible anti-infection option. Therefore, if the *bla*_*NDM–*1_-and *mcr-9-*co-harboring plasmid becomes widely spread among Enterobacteriales, it will likely bring more serious adverse consequences. Therefore, it is imperative to establish a molecular screening method for *mcr-9* to facilitate its rapid and accurate detection.

## Conclusion

Here, we reported the *bla*_*NDM–*1_- and *mcr-9-*co-encoding transferable IncHI2/HI2A plasmid, pK710429_2, obtained from the ST1308 MDR clinical *K. pneumoniae* strain, KPN710429, recovered in China. WGS and comparative genomics assays revealed that plasmid pK710429_2 could be a hybrid plasmid, formed by a Tn*6696*-like *bla*_*NDM–*1_ region inserting into a *mcr-9*-positive-IncHI2/HI2A plasmid, indicating that horizontal gene transfer events play a key role in the plasmid evolution. Although pK710429_2 lacked *qseC-qseB*, the *mcr-9* showed increased expression when induced with colistin, leading to colistin resistance in KPN710429. This finding indicated that the co-transfer of carbapenemase-encoding gene and *mcr-9* in clinically important pathogens poses a high risk to the clinical treatment. Effective surveillance is needed to assess the prevalence of the plasmid co-harboring carbapenemase-encoding gene and *mcr-9*.

## Data Availability Statement

The datasets presented in this study can be found in online repositories. The names of the repository/repositories and accession number(s) can be found in the article/Supplementary Material.

## Author Contributions

XiL, QZ, and JL conceived and designed the experiments. ZL, XH, and XX performed the experiments. WC, XinL, and YL analyzed the data. ZL, XX, and XiL wrote the manuscript. All authors read and approved the final manuscript.

## Conflict of Interest

The authors declare that the research was conducted in the absence of any commercial or financial relationships that could be construed as a potential conflict of interest.

## Publisher’s Note

All claims expressed in this article are solely those of the authors and do not necessarily represent those of their affiliated organizations, or those of the publisher, the editors and the reviewers. Any product that may be evaluated in this article, or claim that may be made by its manufacturer, is not guaranteed or endorsed by the publisher.

## Footnotes

1
http://www.mgc.ac.cn/VFs/

2
https://cge.cbs.dtu.dk/services/ResFinder/

3
https://kaptive-web.erc.monash.edu/

4
https://cge.cbs.dtu.dk/services/PlasmidFinder/

5
https://cge.cbs.dtu.dk/services/pMLST/

## Supplementary Material

Supplementary Figure 1 Serum-killing assays of the tested and references strains. Data are presented as the mean ± standard error, and lg-transformed values were utilized to normalize the data (*n* = 3 for each isolate). KPN54798*^H–ctrl^*, KPN710429, and KPN49*^L–ctrl^* were serum resistant (grade 6), serum intermediate sensitive (grade 3), and serum sensitive (grade 1), respectively.

Supplementary Figure 2 Alignment analysis of the *mgrB* gene sequences between the original KPN710429 strain and the three induced strains. All four *mgrB* gene sequences were identical, and no mismatch was detected.
