# Supplementary material for: Co-occurrence of blaNDM–1 and mcr-9 in a Conjugative IncHI2/HI2A Plasmid From a Bloodstream Infection-Causing Carbapenem-Resistant Klebsiella pneumoniae
Source: Front Microbiol. 2021 Nov 30;12:756201. doi: 10.3389/fmicb.2021.756201 (PMC8701513; doi:10.3389/fmicb.2021.756201)
Supplement: Supplementary file 1 [file Table_1.DOCX]

**TABLE S1 Oligonucleotide sequences of the primers used in this study**

| Primer name | Sequence (5′-3′) | Product size (bp) | Application |
| --- | --- | --- | --- |
| *mcr-9* | Forward, TTCCCTTTGTTCTGGTTG  Reverse, GCAGGTAATAAGTCGGTC | 1011 | PCR |
| *bla*_NDM-1_ | Forward, GCAGCTTGTCGGCCATGCGGGC  Reverse, GGTCGCGAAGCTGAGCACCGCAT | 782 | PCR |
| *mgrB* | Forward, ACCACCTCAAAGAGAAGGCGTT  Reverse, GGCGTGATTTTGACACGAACAC | 347 | PCR |
| *rpoB* | Forward, CGCGTATGTCCGATCGAAA  Reverse, GCGTCTCAAGGAAGCCATATTC | 100 | qRT-PCR |
| *mcr-9* | Forward, CGGTACCGCTACCGCAATAT  Reverse, ATAACAGCGAGACACCGGTT | 131 | qRT-PCR |
| *repA* | Forward, CGGTACCGCTACCGCAATAT  Reverse, ATAACAGCGAGACACCGGTT | 261 | qRT-PCR |
