# Supplementary figures and images for: Co-occurrence of blaNDM–1 and mcr-9 in a Conjugative IncHI2/HI2A Plasmid From a Bloodstream Infection-Causing Carbapenem-Resistant Klebsiella pneumoniae
Source: Front Microbiol. 2021 Nov 30;12:756201. doi: 10.3389/fmicb.2021.756201 (PMC8701513; doi:10.3389/fmicb.2021.756201)

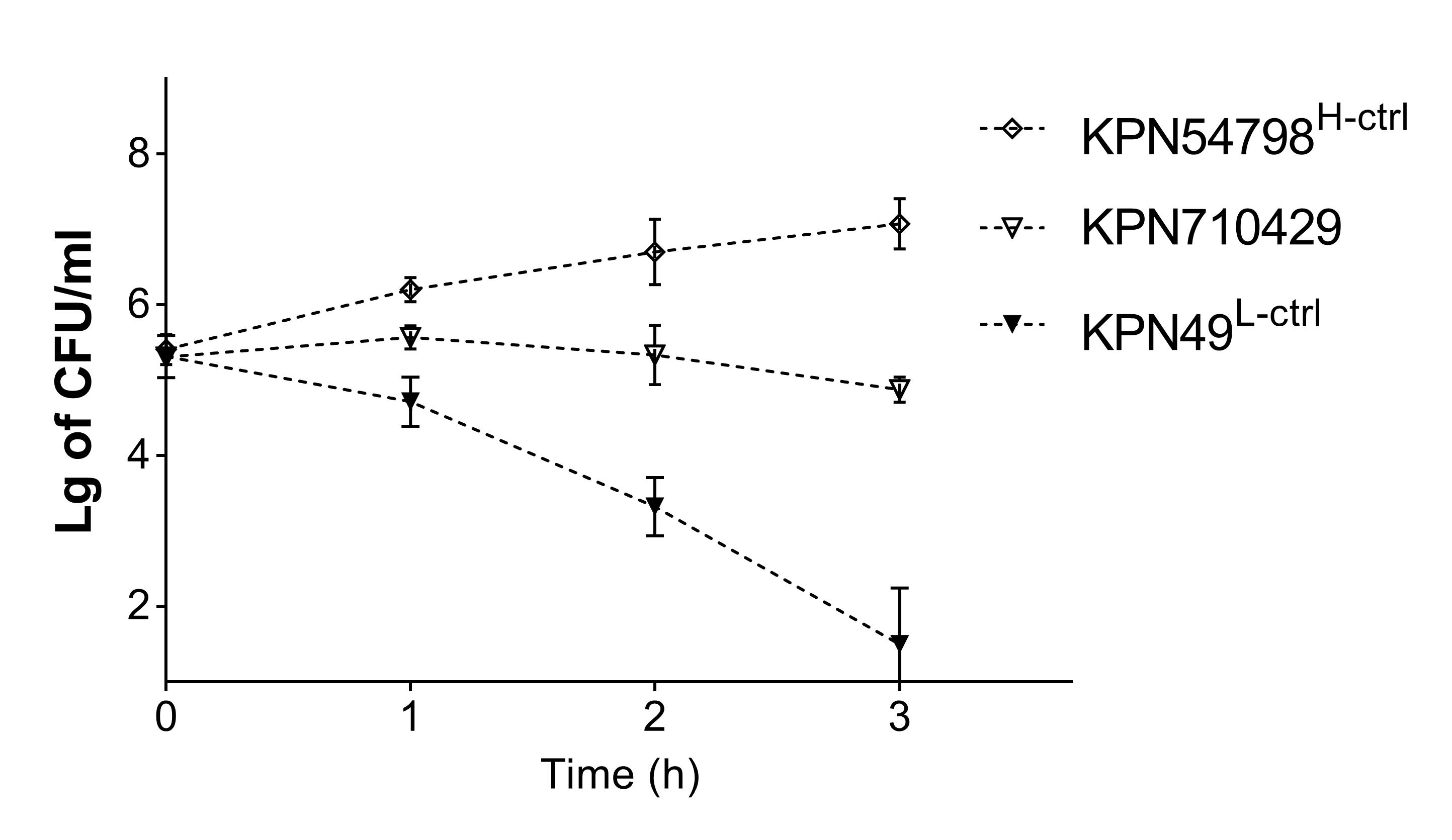

Supplement: Supplementary Figure 1 — Serum-killing assays of the tested and references strains. Data are presented as the mean ± standard error, and lg-transformed values were utilized to normalize the data (n = 3 for each isolate). KPN54798H–ctrl, KPN710429, and KPN49L–ctrl were serum resistant (grade 6), serum intermediate sensitive (grade 3), and serum sensitive (grade 1), respectively. [file Image_1.JPEG]

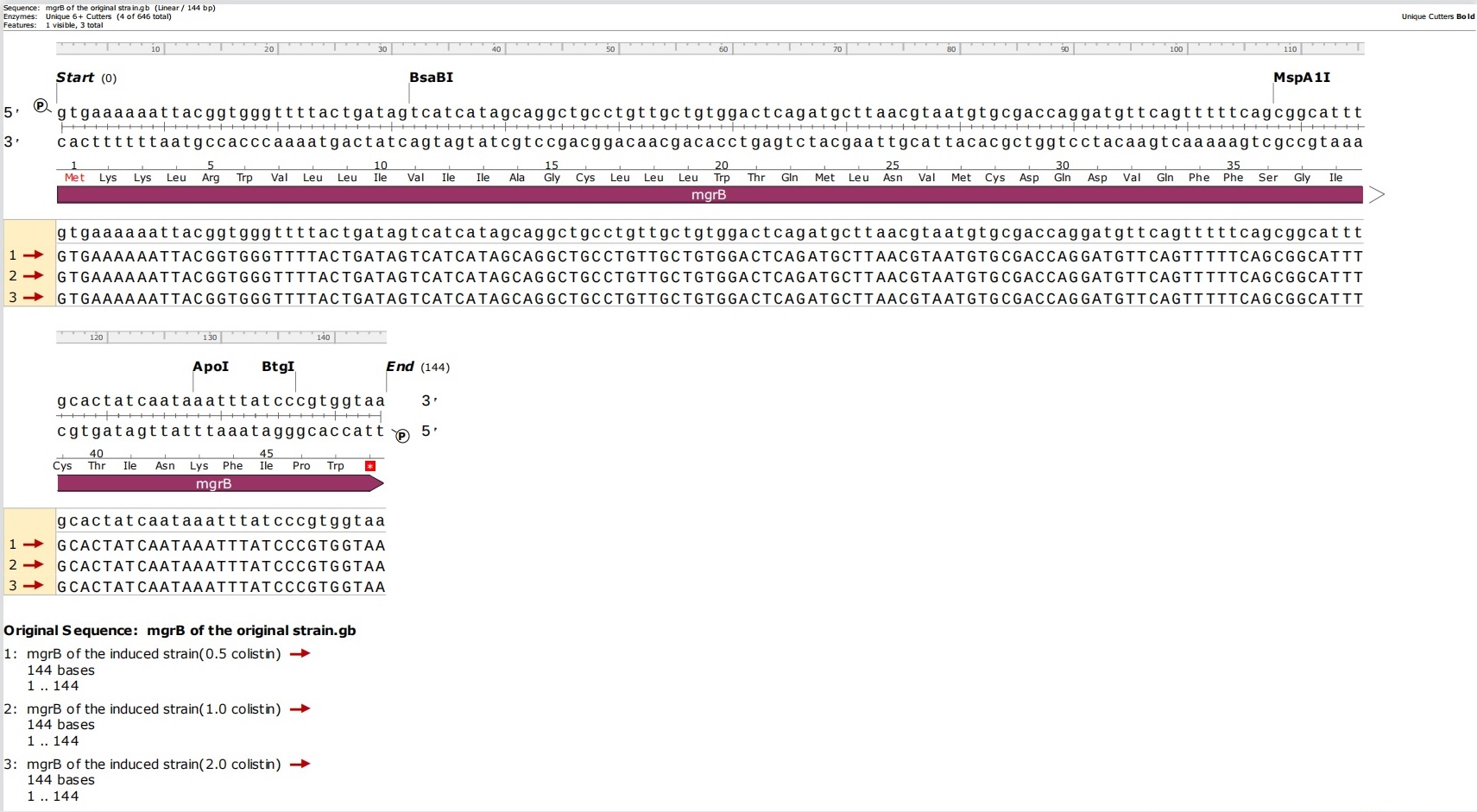

Supplement: Supplementary Figure 2 — Alignment analysis of the mgrB gene sequences between the original KPN710429 strain and the three induced strains. All four mgrB gene sequences were identical, and no mismatch was detected. [file Image_2.JPEG]
